# α-Tocopherol restores semen quality in rats exposed to 2,3,7,8-tetrachlorodibenzo-p-dioxin

**DOI:** 10.14202/vetworld.2022.316-323

**Published:** 2022-02-14

**Authors:** Wurlina Wurlina, Imam Mustofa, Dewa Ketut Meles, Erma Safitri, Suherni Susilowati, Sri Mulyati, Budi Utomo, Suzanita Utama

**Affiliations:** 1 Division of Basic Veterinary Medicine, Faculty of Veterinary Medicine, Universitas Airlangga, Surabaya, Kampus C Mulyorejo, Surabaya 601155, East Java, Indonesia; 2 Division of Veterinary Reproduction, Faculty of Veterinary Medicine, Universitas Airlangga, Surabaya, Kampus C Mulyorejo, Surabaya 601155, East Java, Indonesia

**Keywords:** α-Tocopherol, dioxin, fertility parameters, pollutant, reproductive system

## Abstract

**Background and Aim:**

2,3,7,8-Tetrachlorodibenzo-p-dioxin (TCDD) is a persistent organic pollutant toxic to the human reproductive system. This study aimed to evaluate the effect of α-Tocopherol administration on the male fertility parameters of a rat model exposed to TCDD.

**Materials and Methods:**

Fifty healthy 12-week-old male rats were randomly divided into five groups. Rats in the control group were given corn oil twice daily in 4 h intervals. In the treatment groups, all rats were given TCDD at a dose of 700 ng/kg of body weight (BW)/day for 45 days. Four hours after receiving the TCDD, T0 rats were given corn oil, and T1, T2, and T3 rats were given α-Tocopherol at doses of 77, 140, and 259 mg/kg BW/day, respectively, for 45 days. On day 46, experimental animals were sacrificed to collect blood and testicular samples.

**Results:**

TCDD exposure decreased superoxide dismutase activity, plasma membrane integrity, Leydig cell count, sperm cell count, sperm viability and motility, and increased malondialdehyde levels, serum testosterone levels, and sperm morphological abnormalities. The administration of α-Tocopherol mitigated the effects of TCDD exposure, and the 140 and 259 mg/kg BW/day treatments returned those male fertility parameters to normal levels.

**Conclusion:**

The administration of 140 mg/kg BW/day α-Tocopherol restored male semen quality in rats exposed to TCDD. We found dynamics serum testosterone levels in rats exposed to TCDD that need to be further studied.

## Introduction

2,3,7,8-Tetrachlorodibenzo-p-dioxin (TCDD) is generated as a by-product of industrial processes and uncontrolled waste incinerators [1]. TCDD is a persistent organic pollutant that is toxic, mobile in the environment, and prone to bioaccumulation, representing a global health hazard [2]. More than 90% of human exposure to TCDD is through food, mainly meat and dairy products, fish, and shellfish. TCDD has been reported to be teratogenic, mutagenic, carcinogenic, immunotoxic, and hepatotoxic, affecting the nervous and reproductive systems [3]. Exposure to TCDD induces oxidative stress, characterized by free radicals, and destroys antioxidant defenses in testicular [4]. Testicular cells have antioxidants, namely, enzymatic antioxidants, such as catalase, superoxide dismutase (SOD), and thiol peroxidase, and non-enzymatic antioxidants, such as glutathione. Those endogenous antioxidants are deployed to protect cells from reactive oxygen species (ROS)-induced cellular damage [5]. SOD plays a role in catalyzing superoxide free radicals into oxygen and hydrogen peroxide [6], preventing lipid peroxidation. The primary lipid peroxidation products are lipid hydroperoxides, and malondialdehyde (MDA) is secondary. MDA is a biomarker for lipid peroxidation and an indicator of plasma membrane integrity in testicular cells [7]. However, a higher ROS level imbalance endogen antioxidants, leading to oxidative stress induced in the male reproductive system (5). In the male reproductive system, Leydig cells synthesize testosterone to support spermatogenesis. The absence of testosterone or functional androgen receptors causes male infertility by disturbing spermatogenesis meiosis [8] and reducing sperm count [9]. The number of spermatozoa, sperm viability and motility, and abnormality is crucial for male fertility [10].

Providing antioxidants to scavenge free radicals that can mitigate the effects of oxidative stress on testicular function are needed. α-Tocopherol is a fat-soluble antioxidant, making it more effective as a protector against oxidative stress and preventing lipid peroxide production by scavenging free radicals [11]. Thus, α-Tocopherol is essential in maintaining the physiological integrity of the testes cells, epididymis, and accessory glands that play an essential role in spermatogenesis, spermatozoa maturation [12], and maintenance and survival of spermatids [13]. α-Tocopherol plays a role in protecting against oxidative damage of the testes through its ability to bind to lipid peroxyl radicals before it damages the lipid membrane of the cells [14]. In addition, α-Tocopherol is a non-enzymatic testicular defense system in mitochondria and spermatozoa and can inhibit the peroxidation of testicular damage [15]. TCDD has a high binding affinity to the aryl hydrocarbon receptor (AhR) in the cell cytoplasm, and α-Tocopherol can break this link to rebalance oxidants and antioxidants, mediating TCDD toxicity [16].

The use of α-Tocopherol to counteract the toxic effects of TCDD on the SOD expression, MDA level, sperm plasma membrane integrity (SPMI), Leydig cell number, serum testosterone level, sperm count, sperm viability and motility, and morphological abnormality has not yet been reported. Thus, this study aimed to evaluate the restorative effects of α-Tocopherol on testicular function based on those male fertility parameters using rat (*Rattus norvegicus*) as an animal model.

## Materials and Methods

### Ethical approval

The study was approved by the Animal Care and Use Committee, Airlangga University, Surabaya, Indonesia (No. 267/HRECC.FORM/VI/2020). Experiments have been carried out adequately to minimize pain or discomfort in accordance with the guidelines established by the Institutional Animal Ethics Committee.

### Study period and location

This study was conducted from July 2020 to January 2021. The rats were reared in the Experimental Animal Laboratory, and the other laboratory work was conducted at the Pharmacology Laboratory of Faculty of Veterinary Medicine, Airlangga University.

### TCDD and α-Tocopherol dosages

We administered daily doses of TCDD (Sigma-Aldrich, Darmstadt, Germany) at the rate of 700 ng/kg of body weight (BW), based on a previous study [17]. The α-Tocopherol (Sigma-Aldrich) treatment doses were 77, 140, and 259 mg/kg/day. Corn oil (Mazola^®^, CODAA Switzerland AG) was used as a control and solvent for TCDD and α-Tocopherol [17].

### Treatment of experimental animals

Fifty healthy 12-week-old male rats (200 g) were randomly divided into five groups. Rats in the control group (CG) were given corn oil twice daily in 4 h intervals. In the treatment groups, rats were given 700 ng of TCDD/kg of BW daily. Four hours after the TCDD dose, rats in T0 were given corn oil, and rats in T1, T2, and T3 were given 77, 140, and 259 mg of α-Tocopherol/kg BW, respectively. TCDD and α-Tocopherol were given orally for 45 days, and on day 46, all experimental animals were sacrificed to collect blood and testicular samples.

### SOD, MDA, and testosterone level measurements

The SOD levels in rat testicular tissue were measured using a colorimetric SOD activity assay kit (Sigma-Aldrich, Darmstadt, Germany) at a wavelength of 550 nm [18]. Blood samples were collected from the aortic arch to obtain serum for measuring the MDA and testosterone levels. The MDA levels in serum were measured using the thiobarbituric acid-reactive substance (Sigma-Aldrich) method with an ultraviolet-1601 spectrophotometer at a maximum wavelength of 535 nm [19]. Serum testosterone was measured using a solid-phase competitive chemiluminescence enzyme immunoassay with a testosterone kit (IMMULITE^®^ 1000 total testosterone, Siemens AG, Munich, Germany) [20].

### Evaluation of sperm count, viability, SPMI, motility, and abnormalities

Semen was collected through the cauda epididymis and diluted 200 times with 0.9% NaCl solution. We used a Nikon E200 light microscope (Nikon Corporation, Singapore) for semen evaluation. The sperm count was performed using a Neubauer improved counting chamber (Sigma-Aldrich) at ×100. Spermatozoa viability was assessed through eosin-nigrosin (Sigma-Aldrich) staining on dry swab preparations. Percent viability was determined from 100 spermatozoa in one field of view at ×400 according to the method adopted [21].

SPMI assessment was performed with the hypo-osmotic swelling test. The hypo-osmotic solution was the solution of 7.35 g of sodium citrate 2H_2_O and 13.52 g of fructose dissolved in 1000 mL of distilled water (150 mOsmol of fructose and 150 mOsmol of sodium citrate) (all from Sigma-Aldrich). A 0.1 mL sample was added to 1 mL of hypo-osmotic solution and incubated for 30 min at 37°C. The sperm cells were observed under a light microscope Nikon E200 light microscope (Nikon Corporation, Singapore) at ×400. The intact plasma membrane integrity was indicated by circular tails, whereas damaged plasma membranes were indicated by straight tails [21].

Sperm motility was determined immediately after the semen collection and dilution. The motile spermatozoa percentage was determined from 100 spermatozoa in one field of view at ×400 [22]. The number of morphological abnormalities (head, neck, and tail) in 100 spermatozoa in one field of view was counted on a smear with eosin-nigrosin staining at ×400 [23].

### Leydig cell count

We prepared histological slides with hematoxylin-eosin staining. Leydig cells were counted in five randomly selected seminiferous tubules interspaces, and the mean was calculated. Observations were performed at ×400 using a light microscope equipped with OptiLab Viewer Software Version 2.2. (PT Miconos, Yogyakarta Indonesia) [17].

### Statistical analysis

The results were analyzed using a one-way analysis of variance followed by Tukey’s honestly significant difference test at a 95% confidence level (SPSS Version 23.0) (IBM Corp., NY, USA).

## Results

TCDD exposure decreased the SOD activity, SPMI, Leydig cell count, and sperm cell count, viability, and motility and increased the MDA levels, testosterone levels, and number of abnormal spermatozoa in rats. The administration of α-Tocopherol mitigated the effects of TCDD.

### SOD, MDA levels, and SPMI

Compared with rats in the CG, TCDD exposure (T0) decreased the SOD activity and SPMI (p<0.05) and increased the MDA levels (p<0.05) in rats in the treatment groups. The administration of α-Tocopherol significantly improved the SOD activity, MDA level, and SPMI in T1, T2, and T3 rats compared with those in T0 rats (p<0.05). The doses of 140 (T2) and 259 (T3) mg of α-Tocopherol/kg of BW daily completely mitigated the negative impacts of TCDD exposure, resulting in SOD, MDA, and SPMI measurements comparable (p>0.05) to those of unexposed rats (CG; Table-1).

**Table 1:** Semen superoxide dismutase (SOD) activity, malondialdehyde (MDA) levels, and intact plasma membrane (SPMI) in rats (*Rattus norvegicus*) exposed to 2,3,7,8-tetrachlorodibenzo-p-dioxin (TCDD) and treated with α-Tocopherol.

Group	SOD activity (%)	MDA level (nm/mL)	SPMI
CG	71.08±9.17^b^	3.95±0.74^b^	75.20±4.36^a^
T0	32.64±3.49^a^	9.49±1.78^a^	29.6±5.18^d^
T1	56.23±3.42^c^	3.29±0.34^c^	53.6±6.94^c^
T2	66.04±2.60^b^	4.19±0.55^b^	65.4±7.04^ab^
T3	68.21±6.51^b^	4.55±0.23^b^	71.4±6.25^a^

Different superscripts in the same column show significant differences (p<0.05). Rats in the control group (CG) received 0.5 mL of corn oil twice daily at 4 h intervals for 45 day. Rats in groups T0, T1, T2, and T3 received 700 ng TCDD/kg of body weight (BW) daily, and 4 h later received corn oil, 77, 140, and 259 mg α-Tocopherol/kg BW, respectively, for 45 days

### Leydig cell count, testosterone levels, and sperm count

**Figure-1:**
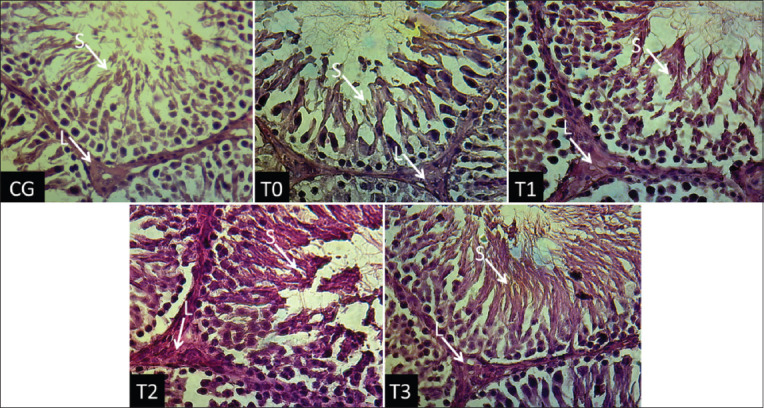
Histologic of rat testicle. L: Leydig cell, S: Spermatozoa. CG: Control group rats, T0, T1, T2, and T3: Rats received 700 ng TCDD/kg BW/day for 45 days, followed by administration of the α-Tocopherol 0, 77, 140, and 259 mg/kg BW/day 4 h later for 45 days (HE staining, ×400).

Table-2 shows that TCDD exposure in rats (T0) caused a 34% decrease (p<0.05) in Leydig cell counts (from 19.14±1.02 in the CG group to 12.72±1.44 in the T0 group), 69% decrease (p<0.01) in sperm cell counts (from 216.60±21.99 in the CG group to 67.40±9.08 in the T0 group), and 266.49% increase (p<0.01) in the testosterone levels (from 37.61±3.06 in the CG group to 137.84±12.42 ng/dL in the T0 group). The administration of α-Tocopherol in T1, T2, and T3 rats increased Leydig and sperm cell counts and decreased the testosterone levels compared with those in T0 rats (p<0.05 for all parameters, except Leydig and sperm cell counts in T1). The doses of 140 (T2) and 259 (T3) mg of α-Tocopherol completely mitigated the negative impacts of TCDD exposure, resulting in similar (p>0.05) Leydig cell count, sperm cell counts and testosterone levels to those of the unexposed rats (CG) (Table-2). Visualization of Leydig cell and population of sperm in each treatment group are shown in Figure-1.

**Table 2:** Leydig cell number, testosterone levels, and sperm count in rats (*Rattus norvegicus*) exposed to 2,3,7,8-tetrachlorodibenzo-p-dioxin (TCDD) and treated with α-Tocopherol.

Group	Leydig cell number	Testosterone levels (ng/dL)	Sperm cell count
CG	19.14±1.02^b^	37.61±3.06^b*^	216.60±21.99^a*^
T0	12.72±1.44^c^	137.84±12.42^a*^	67.40±9.08^c*^
T1	15.04±2.75^c^	30.64±4.46^c^	98.50±13.34^c^
T2	17.89±1.12^b^	35.48±1.89^b^	171.40±30.47^ab^
T3	18.96±2.88^b^	40.92±2.07^b^	206.60±29.27^a^

Different superscripts in the same column show significant differences (p<0.05);

* in the same column show very significant differences (p<0.01). Rats in the control group (CG) received 0.5 mL of corn oil twice daily at 4 h intervals for 45 days. Rats in groups T0, T1, T2, and T3 received 700 ng TCDD/kg of body weight (BW) daily, and 4 h later received corn oil, 77, 140, and 259 mg α-Tocopherol/kg BW, respectively, for 45 days

### Sperm viability, motility, and morphological abnormalities

**Figure-2:**
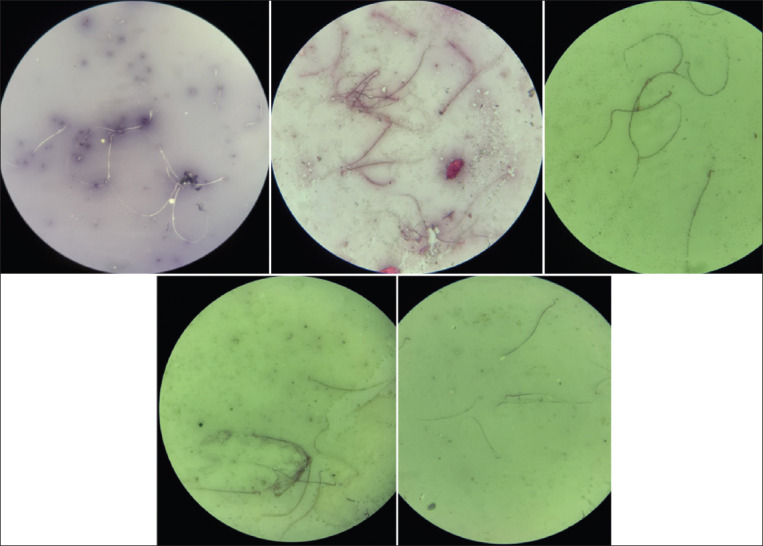
Microscopic examination of rats semen. Alive spermatozoa: Transparent (did not absorb die), dead spermatozoa: Red colored (absorb die), spermatozoa with intact plasma membrane: Coiled tail, spermatozoa with damaged plasma membrane: Straight tail. CG: Control group rats, T0, T1, T2, and T3: Rats received 700 ng TCDD/kg BW/day for 45 days, followed by administration of the α-Tocopherol 0, 77, 140, and 259 mg/kg BW/day 4 h later for 45 days (HE staining, ×400).

TCDD exposure in rats (T0) reduced sperm viability (p<0.05) and motility (p<0.05) and increased the number of sperm morphological abnormalities (p<0.05) compared with those in unexposed rats (CG). The administration of α-Tocopherol significantly increased sperm viability and motility and decreased sperm morphological abnormalities in T1, T2, and T3 rats compared with those in T0 rats (p<0.05). α-Tocopherol completely mitigated the effects of TCDD in T2 and T3 rats, and no significant differences in sperm viability, motility, and morphological abnormalities (p>0.05) were observed compared with those in the unexposed rats (CG; Table-3, Figure-2).

**Table 3:** Sperm viability, motility, and morphologic abnormality in rats (*Rattus norvegicus*) exposed to 2,3,7,8-tetrachlorodibenzo-p-dioxin (TCDD) and treated with α-Tocopherol.

Group	Viability	Motility	Abnormality
CG	87.70±3.97^a^	85.10±4.74^a^	13.50±2.91^d^
T0	35.20±5.05^d^	31.50±7.87^c^	44.80±6.89^a^
T1	67.80±6.62^c^	64.20±6.23^b^	30.10±5.21^b^
T2	75.50±6.09^ab^	72.60±5.60^ab^	20.50±3.17^cd^
T3	84.10±3.81^a^	81.20±6.10^a^	15.20±1.93^d^

Different superscripts in the same column show significant differences (p<0.05); * in the same column show very significant differences (p<0.01). Rats in the control group (CG) received 0.5 mL of corn oil twice daily at 4 h intervals for 45 days. Rats in groups T0, T1, T2, and T3 received 700 ng TCDD/kg of body weight (BW) daily, and 4 h later received corn oil, 77, 140, and 259 mg α-Tocopherol/kg BW, respectively, for 45 days.

## Discussion

TCDD exposure decreased the SOD activity, SPMI, number of Leydig cells, sperm cell count, viability, and motility and increased the MDA levels, serum testosterone, and sperm morphological abnormalities. The administration of α-Tocopherol alleviated the toxic effects of TCDD rat (*R. norvegicus*) as an animal model.

SOD inactivation results in oxidative stress due to excessive ROS generation [24]. We observed increased MDA levels in rats exposed to TCDD (T0), which reflects the level of damage to the plasma membrane attacked by free radicals. When endogenous antioxidants, such as SOD, cannot offset high ROS levels, membrane integrity is lost, and permeability increases. Therefore, SPMI parameters are contrary to the MDA levels, as observed in our experimental results. Polyunsaturated fatty acids in the cell membrane readily accept unpaired electrons from ROS, causing lipid peroxidation [25]. MDA is an end-product of lipid peroxidation; thus, MDA is a biomarker for oxidative stress [7]. In rats treated with α-Tocopherol after TCDD exposure, we observed increases in SOD activity, reaching levels observed in the TCDD unexposed rats. As an antioxidant, α-Tocopherol scavenges excess free radicals in cells, preventing SOD inactivation [26].

In rats, TCDD increases the ROS and MDA levels and disrupts the plasma membranes of testicular cells, including Leydig cells [27]. Excess free radicals generated due to TCDD exposure can directly damage DNA by attacking purine and pyrimidine bases [28] and initiating apoptosis, activating caspase enzymes involved in DNA fragmentation [29], and causing Leydig cell death. α-Tocopherol prevents lipid peroxidation by changing the lipid peroxyl radicals to less reactive and non-destructive [27]. Our findings were consistent with earlier reports that increasing antioxidant levels and the subsequent reduction in the MDA levels [30] increase plasma membrane integrity and prevent Leydig cell apoptosis [17]. The administration of α-Tocopherol in groups T2 (140 mg/kg BW/day) and T3 (259 mg/kg BW/day) maintained the Leydig cell count at normal levels.

The androgen receptor in Leydig cells mediates the maturation of the steroidogenesis pathway [31]. Thus, we would expect a decrease in the Leydig cell count to reduce testosterone levels. Nevertheless, we observed an increase in the serum testosterone levels in rats exposed to TCDD. This can be attributed to a decrease in spermatogenesis resulting from a decrease in Sertoli and spermatogenic cells caused by TCDD exposure. Normal spermatogenesis is supported by Sertoli cells [32]. However, TCDD may increase testicular inflammation by affecting the secretion of pro-inflammatory cytokines in Sertoli cells [33], thereby decreasing Sertoli cell proliferation [34]. TCDD exposure also reduces the spermatogenic staging and number of spermatogenic cells [17]. In this study, TCDD exposure in rats (T0) caused a 34% decrease in Leydig cell counts and a 69% decrease in sperm cell counts. However, there was an increase of 266.49% in testosterone levels compared with those of normal rats (CG). It is unclear why the testosterone level increased, which may be due to a compensatory mechanism. Other possible alternative mechanisms may be as follows. The testosterone levels in the testes of men and rodents are 25-125-fold higher than those present in serum [8]. The unused testosterone for spermatogenesis diffuses into the interstitial capillaries bound rapidly on transport through systemic circulation [35]. As a result of damage to Sertoli and spermatogenic cells due to TCDD exposure, the testosterone required for the process of spermatogenesis is very little. In addition, the unused testosterone in the testes is released into the circulation; thus, the levels of free testosterone in the serum can be very high compared with those in normal rats. This hypothetical opinion must be proven in further studies.

The administration of α-Tocopherol restored Leydig cell function after TCDD exposure through the following mechanisms. First, α-Tocopherol breaks the bond between TCDD and the AhR [16], which regulates cell cycle, proliferation, and differentiation [35]. Second, α-Tocopherol scavenges ROS and modulates the transcriptional regulation of antioxidant enzymes, increasing steroidogenic acute regulatory protein expression for testosterone synthesis [36]. α-Tocopherol also wards off free radicals that affect the converting 17-hydroxyprogesterone and androstenedione to testosterone [37]. The recovery effect of α-Tocopherol on the testosterone levels after TCDD exposure was observed in rats treated with 140 and 259 mg/kg BW/day. Along with the normal return of Sertoli and spermatogenic cell function, more testosterone was used for spermatogenesis, and serum testosterone returned to normal levels.

TCDD damages the membranes of spermatogenic cells and cells supporting spermatogenesis (Sertoli and Leydig cells), resulting in a reduced number of spermatozoa [27]. Excessive ROS formation is associated with a decreased sperm number, sperm motility, morphological abnormalities, and sperm viability. Free radicals can also cause hormonal disturbances and spermatogenesis [38]. Meanwhile, high serum testosterone levels result in reduced spermatogenesis due to negative feedback on the hypothalamus and anterior pituitary to inhibit the release of hormones involved in spermatogenesis [39]. Spermatozoa motility comes from tail movement, closely related to sperm viability and morphology. Energy for the motility of spermatozoa is derived from the breakdown of ATP produced in the mitochondria [40]. The mitochondrial cell membrane is rich in lipids sensitive to free radical attack [41]. Thereby, damage to the mitochondrial DNA is associated with reduced sperm motility [42]. α-Tocopherol can protect the health of the mitochondrial plasma membrane to maintain sperm motility [14]. In our study, α-Tocopherol completely mitigated the effects of TCDD in T2 (140 mg/kg BW/day) and T3 (259 mg/kg BW/day) rats. Our results were consistent with those of Ghafarizadeh *et al*. [43], who showed that α-Tocopherol increases sperm motility and viability *in vitro*.

Semen containing more than 20% of spermatozoa with morphological abnormalities has been shown to reduce fertility [44]. Spermatozoa with abnormal morphology become a source of ROS, especially those containing cytoplasmic remnants, due to the failure of the spermatogenesis process [45]. Sperm structural defects coexist with abnormal nuclear sperm DNA dispersion. Oxidative stress acts as a mediator of damage to the plasma membrane, causing morphological abnormality [46]. The administration of α-Tocopherol prevents the deleterious effects of morphological sperm abnormalities [14]. We observed a significant reduction in abnormalities when α-Tocopherol was administered after TCDD exposure.

We found that α-Tocopherol doses of 140 and 259 mg/kg BW/day effectively restored the SOD activity, SPMI, Leydig cell count, sperm cell count, viability, motility, testosterone levels, reduced the MDA levels, and spermatozoa morphological abnormalities. Sperm quality measurement is not limited to the parameters presented in this study but involves several complex variables. The decreasing sperm viability is due to the death of sperm due to apoptosis and necrosis [47]. Conversely, the changes in sperm quality parameters involve the dynamics of complex endocrine upregulation [38,39]. Thus, there is a need for further studies of α-Tocopherol treated on dioxin-induced male rats by measuring changes in sperm apoptosis and necrosis and the levels of GnRH, FSH, LH, and testosterone in the testicle and serum. Furthermore, the expression of GnRH, FSH, LH receptors, and testosterone in the testicle is interesting to be confirmed.

## Conclusion

TCDD exposure decreased SOD activity, SPMI, number of cells supporting spermatogenesis and increased the MDA and serum testosterone levels. The higher serum testosterone levels may be caused by the release of unused testosterone for the spermatogenesis process to the circulation. The administration of 140 mg/kg BW/day α-Tocopherol mitigated the negative impacts of TCDD exposure and restored the semen quality of rats. We found dynamics serum testosterone levels in rats exposed to TCDD that need to be further studied.

## Authors’ Contributions

WW, IM, DKM, ES, SM, SS, BU, SU. WW and DKM: Compiled ideas and designed the study. WW and SM: Treatment of rats. IM and SU: Measured MDA levels and testosterone concentration. ES and RR: Evaluated SOD expression. SS and BU: Evaluated IPM, sperm viability, motility, and morphologic abnormality. DKM and IM: Statistical analysis and conceived the manuscript. WW, IM, and DKM: Drafted the manuscript. SM, ES, and SU: Critically read and revised the manuscript for intellectual content. All authors read and approved the final manuscript.
